# Development of an Intratumoral Holmium Microsphere Injection Method in Ex Vivo Human Pancreatic Ductal Adenocarcinoma: A Preclinical Feasibility Study

**DOI:** 10.3390/cancers17061028

**Published:** 2025-03-19

**Authors:** Coen Ysbrand Willink, Sjoerd Franciscus Maria Jenniskens, Nienke Johanna Maria Klaassen, Martijn Willem Jan Stommel, Cornelis Johannes Henricus Martinus van Laarhoven, Jurgen J. Fütterer, Johannes Frank Wilhelmus Nijsen

**Affiliations:** 1 Department of Medical Imaging, Radboud University Medical Center, Geert Grooteplein-Zuid 10, Postbox 9101, 6500 HB Nijmegen, The Netherlandsfrank.nijsen@radboudumc.nl (J.F.W.N.); 2 Department of Surgery, Radboud University Medical Center, Geert Grooteplein-Zuid 10, Postbox 9101, 6500 HB Nijmegen, The Netherlands

**Keywords:** pancreatic cancer, local therapy, novel therapy, intratumoral injection, microbrachytherapy, holmium microspheres, fundamental research

## Abstract

Background/Objectives: Patients diagnosed with pancreatic ductal adenocarcinoma (PDAC) have a poor prognosis. Local therapy may enhance tumor control and increase resectability. Intratumoral injection of radioactive holmium-166 microspheres presents a promising and minimally invasive treatment with multimodality imaging capabilities (SPECT, CT, MRI). However, holmium-166 microspheres are not commonly used for intratumoral injections, and PDAC is notorious for its high intratumoral pressure. This study developed an intratumoral injection method with nonradioactive holmium-165 microspheres in ex vivo human PDAC specimens using a novel injection system for suspension homogenization. Methods: An injection system was developed and validated in a laboratory setting. Thereafter, intratumoral injections in surgically removed ex vivo PDACs were performed, and parameters were established to optimize feasibility, defined by the ability to inject and control the microsphere distribution. Also, injection limitations and cutoff values were determined. The distribution was assessed by visual confirmation, CT, MRI, ultrasound, and histopathology. Results: With a validated injection system, intratumoral injections were performed in ten ex vivo PDAC samples. Feasible injection guidelines include but are not limited to ultrasound or CT needle guidance, a maximum injection volume of <20.0% from the tumor volume, ≤3 needle positions, and an injection volume of 0.3–1.0 mL per needle position. Conclusions: Intratumoral injection of holmium-165 microspheres in ex vivo pancreatic ductal adenocarcinoma was feasible with adherence to injection parameters necessary for effective intratumoral deposition and minimal leakage. The injection system and parameters developed here provide a foundation for future studies on holmium-166 microsphere injections in pancreatic cancer patients, with the aim to improve local tumor control as a part of a multimodal therapy.

## Simple Summary

Pancreatic cancer has a poor outlook, and surgery is currently the only potentially curative option. This research explores a new treatment approach using injections of radioactive holmium microspheres directly into tumors, which could improve local tumor control and possibly make more tumors eligible for surgical removal. Researchers tested a system to inject holmium microspheres into surgically removed pancreatic cancer samples to assess how well the microspheres could be injected and spread within the tumor without leaking outside. Through this study, they developed specific guidelines for effective injections. These findings lay the groundwork for future studies of radioactive holmium microsphere injections in patients as part of a combined therapy, which may lead to better outcomes for pancreatic cancer patients.

## 1. Introduction

Pancreatic ductal adenocarcinoma (PDAC) is the most common type of pancreatic cancer. With an overall 5-year survival of 4.2–11% in the past decade, the outcome of patients with PDAC remains dismal [1,2]. Approximately 13–20% of all patients diagnosed with PDAC are eligible for surgical resection, which increases the 5-year survival rate to 17% [3,4,5,6]. The remaining patients have either metastatic disease (50%) or locally advanced pancreatic cancer (LAPC, 30%) [3,4]. Curative treatment for patients with LAPC is only available after a significant tumor response and downstaging, most commonly attempted by induction chemotherapy, which is sufficiently effective in approximately one out of five LAPC patients [7]. Obvious challenges when subjecting (near) terminal patients to extensive treatment are comorbidities, toxicity, and recovery time [8,9]. Although several new minimally invasive therapies have been developed and studied, oncological outcomes have hardly improved [10,11].

Minimally invasive delivery of an anticancer substance by direct intratumoral injection may contribute to local tumor control of LAPC [11]. Intratumoral injection, also called interstitial injection, involves the placement of a needle not in a blood vessel or duct, but directly in the tumorous tissue, and the needle is then used to deliver a substance between the tumor cells [11]. When radioactive microparticles are injected, this therapy is called (micro)brachytherapy. The microparticles act as an implant and are fixated within the tissue after the radiation has decayed [12]. Local irradiation causes a high local tumor dose without damaging nearby vital structures. Additionally, the cytokines induced by the local apoptosis and necrosis might drain towards the tumor-draining lymph nodes and activate a broader immunogenic antitumor effect [13]. Minimally invasive microbrachytherapy can potentially reduce the tumor burden, prolong survival, and cause tumor downstaging, and therefore may increase surgical resection rates [14].

The potential of microbrachytherapy was first seen in the OncoPaC-1 trial, showing successful endoscopic ultrasound (EUS)-guided intratumoral injection of microparticles incorporating radioactive phosphorus-32 (^32^P) in 42 patients with LAPC [14]. A local disease control rate (RECIST 1.1) of 82% (95% confidence interval 68.6–90.9%) after 16 weeks and a median overall survival of 15.5 months was achieved [14]. A disadvantage of ^32^P is that it is a pure beta (β^−^) emitter (characteristics are described in Table 1), which limits imaging of the administered microparticles to low-resolution Bremsstrahlung single-photon emission computed tomography (bSPECT). This hinders accurate assessment of therapy distribution after injection and makes it impossible to assess or adjust therapy distribution during intervention.

Other microparticles eligible for intratumoral injection in LAPC are holmium-166 (^166^Ho) incorporated poly L-lactic acid microspheres (^166^HoMSs). Holmium-166 is a radioactive β^−^ emitting isotope similar to ^32^P, as shown in Table 1. However, ^166^Ho has a shorter half-life, and besides β^−^ also emits low-energetic gamma radiation (γ) with an energy of 80.6 KeV. The γ radiation makes ^166^Ho eligible for conventional SPECT imaging with a higher resolution than bSPECT. Moreover, since ^166^Ho has a high atomic mass (165.9 u) and makes up approximately 19% of the mass of the microspheres, it causes attenuation of X-rays, which makes the microspheres hyperdense on computerized tomography (CT) [15]. Owing to the paramagnetic properties of holmium, the microspheres are also imageable by magnetic resonance imaging (MRI) [16]. For research purposes, holmium-165 microspheres (^165^HoMSs) are often utilized. These microspheres are identical to ^166^HoMSs with the exception of containing a stable isotope and therefore not emitting any β^−^ or γ radiation. Although ^165^HoMSs may not serve a therapeutic purpose, they can still be analyzed using CT or MRI imaging.

^166^HoMSs are commonly used for transarterial radioembolization (TARE) of primary and secondary hepatic malignancies [17,18]. For TARE, it serves as a feasible and safe treatment with beneficial clinical outcomes and can achieve treatment-free intervals or bridging-to-transplant [19,20]. TARE can only be applied in the liver because of the unique dual blood supply, with the tumor receiving primarily arterial blood and the liver parenchyma portal-venous blood [21]. However, intratumoral ^166^HoMS injection has also been previously studied, for example, in feline patients with oral squamous cell carcinoma [22]. Intratumoral injection of ^166^HoMSs resulted in minimal side effects, a complete response or partial response with subsequent marginal resection in 6 out of 11 (55%) feline patients, and more than doubled the survival time in responders vs. nonresponders [22]. Moreover, intratumoral ^166^HoMS injection was performed in three human patients suffering from head and neck cancer [23]. Even though no adverse events were observed, the therapeutic effect was minimal due to the low tumor dose and technical obstacles that further limited the delivered tumor dose. The microspheres are known to settle quickly in a suspension because the density is greater than that of the injection fluid. This caused heterogeneous suspensions in the syringe during injection, resulting in needle blockage and a varying injected ^166^HoMS concentration between 17.8 and 84.3% of the initial concentration [23].

This study aimed to develop an intratumoral injection method in ex vivo human pancreatic ductal adenocarcinomas using nonradioactive ^165^HoMSs. First, an administration system that enables continuous motion of the microsphere suspension was developed to gain more control of the administered microsphere concentration. This system was tested to establish injection system parameters (ISPs), which were then validated in a laboratory setting. Next, the validated ISPs were translated to intratumoral injections in ten ex vivo pancreatic ductal adenocarcinoma samples obtained from patients after surgical resection to establish intratumoral injection parameters (IIPs) and determine the feasibility of the developed method.

## 2. Materials and Methods

In this study, a new intratumoral injection system was designed, and ISPs were validated for homogeneous ^165^HoMS injection and thereafter applied to ten surgically removed human pancreatic ductal adenocarcinoma samples. IIPs were established to determine the optimal intratumoral injection control and distribution and to find the cutoff values and limitations.

### 2.1. Injection System Design

**Figure 1:**
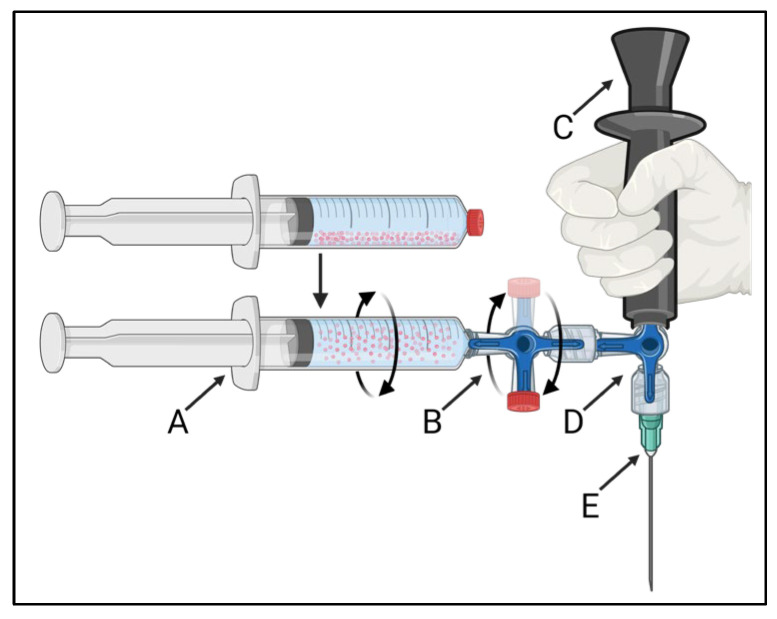
A schematic overview of the 360° syringe-rotating injection system that was used to create a homogeneous ^165^HoMS suspension and to inject constant concentrations. The injection system consists of a ^165^HoMS syringe (A) connected to a 360° rotating 3-way stopcock (B), enabling the syringe to be manually rotated over its long axis. The rotatable 3-way stopcock was connected to a second 3-way stopcock (D) with a handgrip (C) and needle attached (E) perpendicular to the ^165^HoMS syringe. Created with BioRender.com.

The developed injection system is schematically presented in Figure 1. The ^165^HoMS syringe (Figure 1A) was connected to a 3-way stopcock with a single 360^°^ rotating male Luer-lock connection (Figure 1B: CODAN, Lensahn, Germany), enabling the 3-way stopcock and ^165^HoMS syringe to be manually rotated over its long axis. Opposite the ^165^HoMS syringe, the rotatable 3-way stopcock was connected to a second 3-way stopcock (Figure 1D) with a handgrip (Figure 1C) and needle (Figure 1E) attached perpendicular to the long axis of the ^165^HoMS syringe. Needles of 18–21 Gauge (G) and 50–150 mm were used. Manual syringe rotation was performed with the aim of creating a homogeneous ^165^HoMS suspension up to the moment of injection to enable the injection of constant ^165^HoMS concentrations. A plastic extension tube (21 G, 10–30 cm; CODAN, Lensahn, Germany) could be attached between the second 3-way stopcock (Figure 1D) and the needle (Figure 1E) to enable more freedom for needle placement. Air was removed from the injection system by priming the system with either demi-water for validation testing or saline for ex vivo intratumoral injection before the ^165^HoMS syringe was connected. All the components were connected by a Luer-lock to withstand internal pressures and prevent suspension leakage.

Initial laboratory testing was performed to assess the feasibility of the injection system and visual effectiveness in suspension homogenization with multiple ISPs, including the syringe volume (1.0–10.0 mL), rotation time (~1–120 s), rotation speed (30–120 rotations per minute; RPM), injection speed (0.05–1.0 mL/s), minimal injection volume (0.1–1.0 mL), and injection system components. The most promising ISPs from the initial laboratory tests were then validated.

### 2.2. Injection System Validation Testing

During injection system validation, a set of ISPs defined during initial laboratory testing was used to validate a homogeneous and constant ^165^HoMS concentration injection. Injection system validation was performed by injecting ^165^HoMS suspensions in glass validation vials, evaporating the injection fluid, and weighing the residual ^165^HoMSs to establish injection recoveries (R_HoMS_ in percentages) and consistency between injections, as described below.

#### 2.2.1. Microsphere Preparation

In this study, dry nonradioactive holmium-165 poly L-lactic acid microspheres (^165^HoMSs) were used (Quirem Medical, Deventer, The Netherlands). The ^165^HoMS values were within the specifications of radioactive microspheres (QuiremSpheres^®^), with a diameter of 30 μm (±5 μm) and 19.0–20.0 *w*/*w*% ^165^Ho. The ^165^HoMSs were suspended in a water-based injection fluid with an isotonic phosphate buffer (116 mM, pH 7.4) and 0.1–2.0% Pluronic (Quirem Medical, Deventer, The Netherlands), unless stated otherwise.

#### 2.2.2. Laboratory Validation Testing

For validation assessment, a predetermined concentration aimed at 20 milligrams of ^165^HoMS per milligram of injection fluid (mg/mg) in 3.0 mL syringes (Becton, Dickinson and Company, Franklin Lakes, NJ, USA) was prepared and weighed to determine the prepared ^165^HoMS concentration in the injection fluid (C_syringe_ in mg/mg). Each syringe was connected to its own primed injection system. After manual rotation, the suspension from each syringe was manually injected in 0.5 mL fractions into six separate 20.0 mL glass validation vials (PerkinElmer, Drachten, The Netherlands). After a syringe was emptied, the injection system was flushed repeatedly with 6.0 mL of demi-water to remove agglomerated microspheres. The agglomerated microspheres were collected in glass flush vials. Control vials were prepared in triplicate with 1.0 mL of injection fluid and 1.0 mL of demi-water. After all the injections were performed, the injection vials and control vials were placed in a 50 °C stove for at least 48 h to evaporate all the liquid. All vials were weighed in mg on a microbalance (XPE105, d = 0.01 mg; Mettler Toledo, Tiel, The Netherlands) when empty (M_empty_), full (M_full_), and after evaporation (M_evaporation_). With these measurements, the mass of the vial’s contents (indicated by M_c_) and the residue after evaporation (indicated by M_r_) were also determined in mg (Equations (1) and (2)).

(1) $$M_{c}=M_{\mathrm{full}}-M_{\mathrm{empty}}$$

(2) $$M_{r}=M_{\mathrm{evaporation}}-M_{\mathrm{empty}}$$

The concentration of the residue (C_r_ in mg/mg) per vial was determined as shown in Equation (3).

(3) $$C_{r}=\frac{M_{r}}{M_{c}}$$

The concentration of ^165^HoMSs (C_HoMS_ in mg/mg) in the flush and validation vials was determined by extracting the mean concentration residue ($\bar{x}$C_r−control_) from the demi-water or injection fluid control vials from the concentration residue (C_r_) of the flush or validation vial, respectively, as shown in Equation (4).

(4) $$C_{HoMS}=C_{r}-\bar{x}C_{r-\mathrm{control}}$$

The recovered ^165^HoMS concentration (R_HoMS_, percentages) from the flush (R_HoMS−flush_) and validation vials (R_HoMS−injection_) was determined by dividing the concentration of ^165^HoMSs (C_HoMS_) in the vials by the concentration of ^165^HoMSs in the syringe (C_syringe_), as shown in Equation (5).

(5) $$R_{HoMS}=\frac{C_{HoMS}}{C_{\mathrm{syringe}}}*100\%$$

A maximum range of 20% for the R_HoMS−injection_ (median ±20%) was considered an improvement over previous intratumoral injection results, where injection ranges of up to 33.3% were observed [23].

### 2.3. Ex Vivo Human Pancreatic Ductal Adenocarcinoma

This study was approved by the Medical Ethics Assessment Committee (METC) Oost-Nederland (CMO 2019-5578) and was not subjected to the Law for Medical Research involving Human Subjects. Patients were required to provide informed consent before undergoing surgery to approve the use of medical information, provide consent for publication, and donate resected tissue for research.

Patients older than 18 years with pathologically proven PDAC and a single lesion eligible for surgical resection via pancreatoduodenectomy or distal or total pancreatectomy were eligible for tissue donation. No distinction was made between the presence or type of neoadjuvant therapy. Patients with PDAC < 10 mm in largest diameter on diagnostic contrast-enhanced CT (CECT) were excluded. Tumor volumes were assessed via manual 3D segmentation of diagnostic CECT.

Immediately following surgical resection, the sample was registered and examined for study approval by the Department of Pathology. Metal clips, staples, or stents that could intervene with CT or MRI were removed. Clinical imaging was introduced before, during, and/or after injection with CT (Aquilion, Canon Medical Systems Europe B.V., Zoetermeer, The Netherlands), MRI (Skyra 3T, Siemens Healthineers, Den Hague, The Netherlands), and/or ultrasound (US; Xario 200, Canon Medical Systems Europe B.V.; Zoetermeer, The Netherlands). The imaging parameters used are presented in Table S1. After surgery, no study-related follow-up was performed on the patients.

The evaluated IIPs during ex vivo intratumoral injection included the total injection volume (0.2–4.5 mL), total injection volume with respect to the tumor volume (8–83%), injection volume per deposit (0.1–1.0 mL), number of needle passes (1–13), number of ^165^HoMS deposits (1–45), ^165^HoMS concentration (5.0–50.0 mg/mL), and type of image guidance (none, US, CT). All the injections were performed by, or with the supervision of, an interventional radiologist (S.F.M.J.). After injection, all the samples were fixed with 4% formaldehyde within 4 h to prevent decomposition of the autolytic pancreatic parenchyma. After at least 48 h of fixation, the tissues were sliced, histologically stained (hematoxylin–eosin), and analyzed by the Department of Pathology.

### 2.4. Outcome Measures

Injection feasibility was achieved if intratumoral ^165^HoMS deposition and distribution were observed and visualized via CT and/or MRI. Extratumoral ^165^HoMS leakage is defined as extratumoral ^165^HoMS deposition on CT and/or MRI or by visual confirmation of fluid leakage. Feasibility was pursued by adjusting IIPs with an iteration-based approach to find the optimal intratumoral injection control and distribution. Note that IIPs were also adjusted to find feasibility limitations and cutoff values. The feasibility outcomes included intratumoral microsphere deposition (yes/no), extratumoral microsphere leakage (yes/no), injection observations (descriptive), and type of image guidance. Furthermore, outcomes included advised ISPs (syringe volume, rotation time, rotation speed, flush volume, minimal injection volume, and injection speed) and advised IIPs (tumor volume, injection volume, syringe angle, needle insertions, deposits, volume per deposit, ^165^HoMS concentration, needle diameter, and needle length).

#### Data Processing

Because of the iterative-based study approach, most findings are descriptive or measurements. All reported measurements are described as medians (ranges) unless stated otherwise. Graphs were created in Microsoft Office 365, Excel. MRI and CT processing was performed with RadiAnt DICOM viewer (version 2021.2.2), Q-Suite (version 2.1), and the open-source software 3D Slicer (version 4.11–5.6) [24].

## 3. Results

### 3.1. Injection System Development

Initial laboratory testing resulted in the development of the injection system described previously in Section 2.1, and in the ISPs described in Table 2. A more extensive description of the findings during injection system development is shown in Appendix A.

### 3.2. Injection System Validation

**Figure 2:**
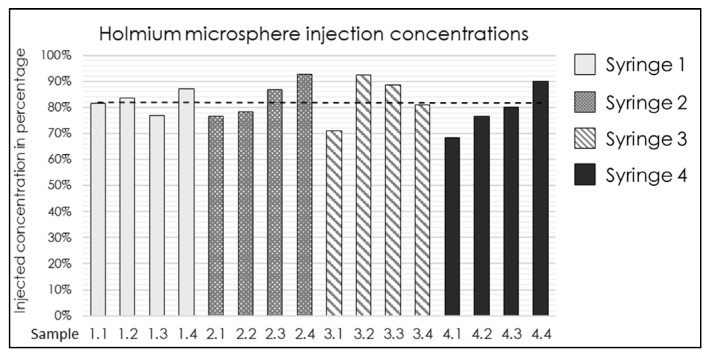
Bar graph of the ^165^HoMS concentration recovered (R_HoMS-injection_) after injection in 16 samples of 0.5 mL compared with the prepared syringe concentration. The black dotted line indicates a median recovered concentration of 81% (range: 69–93%).

The established injection system and determined ISPs (Table 2) were validated. Four syringes were prepared with a median ^165^HoMS concentration of 17.4 mg/mL (range 16.7–18.6). An overall R_HoMS_ of 65% (range 63–68%) was injected for the complete 3.0 mL syringe. The flush was the first 1.0 mL ejected from the injection system and resulted in a lower R_HoMS−flush_ of 44.1% (range 34.7–48.8%). The remaining 2.0 mL resulted in a R_HoMS−injection_ of 81% (range 69–93%); see Figure 2. The R_HoMS−injection_ was applied for ex vivo intratumoral injections.

### 3.3. Ex Vivo Intratumoral Injection

Overall, ten ex vivo PDAC tumor samples were injected and analyzed. The tumor volume on diagnostic CECT ranged from 2.5 to 15.6 mL, with a median of 6.9 mL. A sample containing the tumor often includes healthy pancreatic parenchyma, (part of) the duodenum, resected vasculature, and, sometimes, the spleen, clips, staples, stents, or stitches. The established IIPs based on ex vivo intratumoral injection are presented in Table 3. The most notable results are described below. An overview of all the tissue samples and injection results is presented in Table 4.

#### 3.3.1. Image Guidance

**Figure 3:**
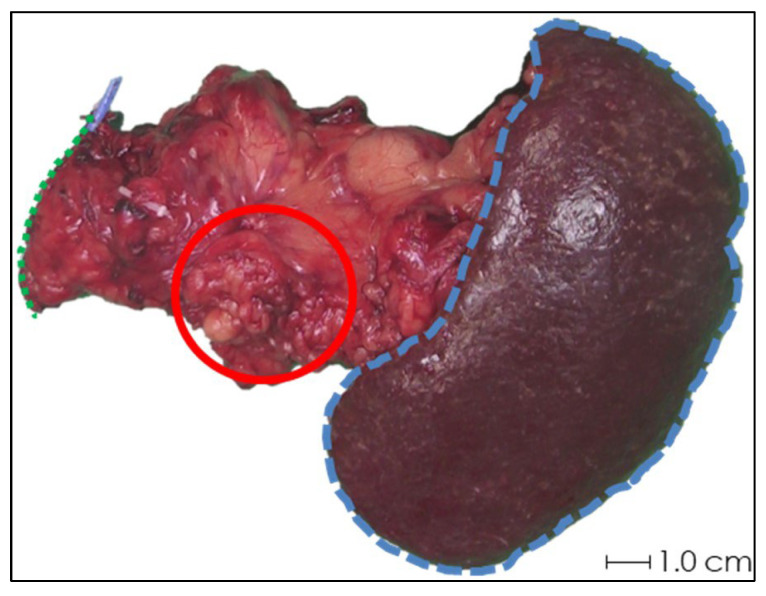
Photograph of the anterior side of ex vivo tissue sample 1 after pancreatic tail resection with splenectomy. Schematic estimations of the pancreatic resection surface (green dotted line), spleen (blue striped line), and rough approximation of the tumor (red circle) are shown. Accurate identification of the tumor by visual inspection was not feasible, and palpation was not sufficient for needle depth estimation.

**Figure 4:**
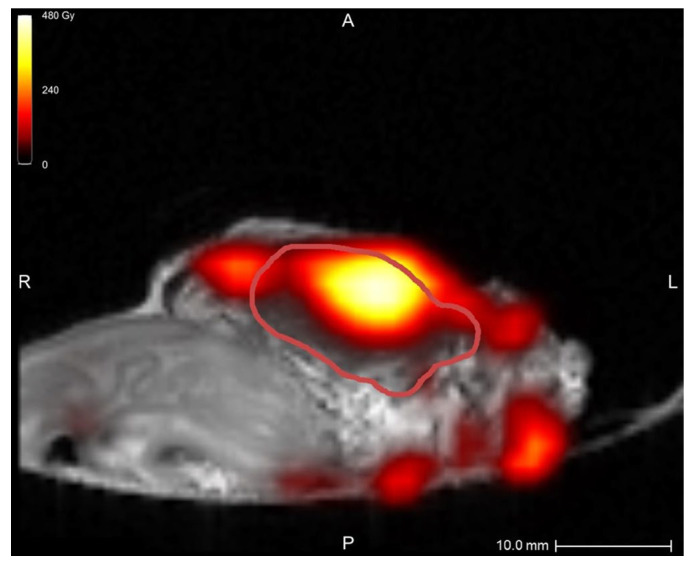
MRI quantification after nonradioactive intratumoral ^165^HoMS injection in sample 5, with a theoretical specific activity of 12.00 MBq/mg ^165^HoMS. The red line delineates the tumor. The highest dose, at the location of the ^165^HoMS deposition, was observed within the tumor. Multiple MRI artifacts caused inaccurate dose estimations surrounding the tissue, which were created by air and heterogeneous tissues. This figure was created in Q-Suite (version 2.1).

**Figure 5:**
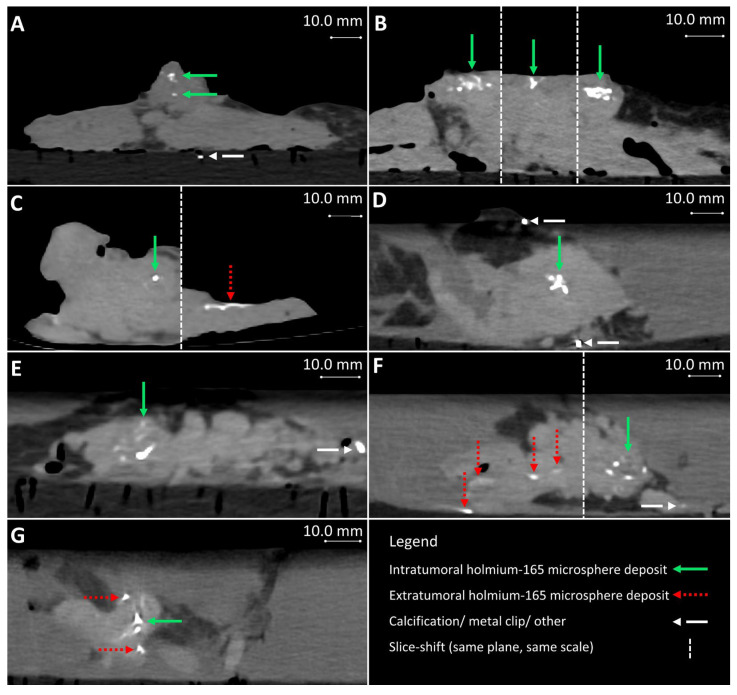
Post-injection CT from samples 4–10 (**A**–**G**), respectively. (**B**,**C**,**F**) contain multiple slices of the same plane and scale. Extratumoral holmium-165 microsphere deposits occurred and are visible in images (**C**,**F**,**G**), for samples 6, 9, and 10, respectively.

**Figure 6:**
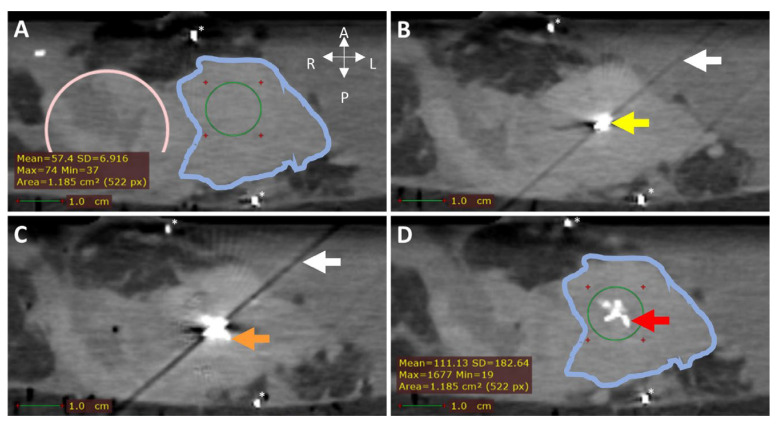
CT (0.5 mm isotropic) of intratumoral ^165^HoMS injection in ex vivo pancreatic cancer sample 7. (**A**) CT before ^165^HoMS injection showing the sample anterior (**left**), posterior (**right**) (white arrows), duodenum (pink circle), tumor border approximation (blue contour), and HU values of interest (**bottom left**) in the region of interest (ROI; green circle). (**B**) CT with a 20G needle in situ. The needle tip is in the viewed slice, causing the hyperdense artifact (yellow arrow) and the diagonal shading artifact (white arrow in **B**,**C**). (**C**) CT scan directly following the injection of 11.5 mg of ^165^HoMSs in 0.7 mL suspension with the needle in situ. Deposition of ^165^HoMSs around the needle tip causes the hyperdense artifact to increase in size (orange arrow). (**D**) CT scan after needle extraction with ^165^HoMSs (red arrow) visible as hyperdense area in the center of the tumor (blue contour); HU values of interest (**bottom left**) in the region of interest (ROI; green circle). * Calcifications.

In the first two samples, periprocedural needle guidance was visual and performed using tumor palpation without image guidance. On the tissue surface, no visual distinction could be made between healthy pancreatic parenchyma and tumor (see Figure 3). Distinguishing between harder and dense tumor tissue and softer, healthy pancreatic parenchyma was possible via palpation. Estimating needle depth by palpation was more challenging, and multiple needle overshoots occurred in samples 1 and 2. Thereafter, US was introduced for periprocedural image guidance of the needle. In samples 3–10, no needle overshoots were observed. Owing to the magnetic and spatial limitations of MRI, it could not be used for periprocedural needle guidance. Additionally, heterogeneous tissues in and around the tumor, air in the samples, and a low spatial resolution made MRI ineligible for ^165^HoMS distribution assessment (see Figure 4). In samples 4–10, ^165^HoMS deposits became clearly visible on post-injection CT because of an increased prepared ^165^HoMS concentration. An overview is presented in Figure 5. For samples 6 to 10, periprocedural CT was added for needle guidance and periprocedural ^165^HoMS visualization (see Figure 6). However, needle guidance by CT was challenging since the ex vivo tissues were not perfused, and therefore CT contrast was unavailable.

#### 3.3.2. Injection Volume, Concentration, and Deposits

The total injection volume per sample ranged from 0.2 to 4.5 mL, with a median injection volume of 1.9 mL. When injection volumes > 2.0 mL were needed, multiple ^165^HoMS syringes could be prepared and replaced between injections. The injection volumes with respect to the tumor volume ranged from 8 to 83%, with a median of 19%. The injection concentration did not affect feasibility during injection, but higher concentrations did result in increased Hounsfield units (HUs) of the ^165^HoMS deposits, and thus a better contrast-to-background ratio on CT [15]. When the prepared ^165^HoMS concentration was ≥10.0 mg/mL (samples 4–10), ^165^HoMS deposits became clearly disguisable on post-injection CT, as presented in Figure 5. On MRI, highly concentrated ^165^HoMS deposits cause large susceptibility artifacts, also called signal voids, which cause an underestimation of ^165^HoMSs when quantified. The number of deposits per sample ranged between 1 and 45, with a median of 2 deposits per sample. The first sample had 45 deposits, which showed severe leakage due to needle tract contact and needle overshoot through the tumor. All remaining samples had between one and five deposits. The injection volume per deposit ranged from 0.1 to 1.0 mL.

#### 3.3.3. Leakage

**Figure 7:**
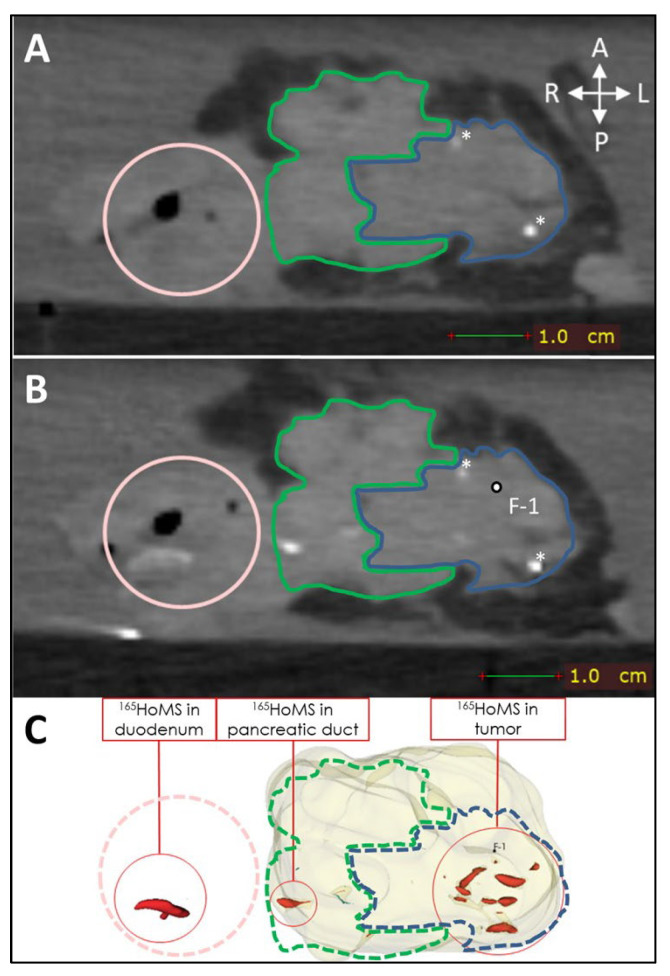
CT (0.5 mm isotropic) of intratumoral ^165^HoMS injection in ex vivo pancreatic cancer sample 9 (vertically aligned). (**A**) CT before ^165^HoMS injection showing the patient’s anterior (**left**), posterior (**right**) (white arrows), duodenum (pink circle), tumor border approximation (blue contour), and healthy pancreas border approximation (green contour). (**B**) CT scan directly following the injection of 13.4 mg of ^165^HoMSs in a 0.7 mL suspension with the needle removed. Leakage of the ^165^HoMSs occurred from the injection location (F-1, out of plane) through the pancreatic duct toward the duodenum. (**C**) Three-dimensional segmentation of the pancreatic head (beige), including the healthy pancreas and tumor, and the ^165^HoMSs (red).

Leakage of needle tracts was observed when two or more needle tracts touched or crossed paths, which occurred only in sample 1. Leakage due to backflow was observed once during injection in a tumor region with high interstitial pressure. Manual injection allowed haptic feedback on injection resistance, which could be used to estimate the risk of injection in a duct or vessel when low resistance was felt or the risk of needle tract leakage when high resistance was felt to due high interstitial pressure. High interstitial pressure was observed multiple times; however, leakage could easily be averted by slightly changing the needle tip position and continuing the injection when normal resistance was felt. Extratumoral leakage, other than leakage by needle tracts, was not observed when the injection volume remained below 20% of the total tumor volume and when needle placement was central in the tumor as confirmed by US. US was used to identify vital structures such as major blood vessels and the pancreatic duct. In the tumor, the pancreatic duct was unidentifiable via imaging due to complete obstruction. Nevertheless, in sample 9, leakage through the pancreatic duct toward the duodenum was observed on post-injection CT without an indication of hitting the pancreatic duct or injecting into it (see Figure 7).

#### 3.3.4. Needle Blockage

For sample 7, the ^165^HoMS syringe was tilted approximately 30 degrees vertically due to a lack of space for the injection system. This caused the ^165^HoMSs to agglomerate in the syringe tip. During injection, a needle blockage was observed after 0.7 mL. This block could be resolved only by extracting the needle from the tissue. To prevent space limitations for the injection system, tubing was added between the injection system and the needle as described in Section 2.1. Furthermore, an unresolvable needle block never occurred with the ^165^HoMS syringe in a near-horizontal position (0–10 degrees).

#### 3.3.5. Histopathology

**Figure 8:**
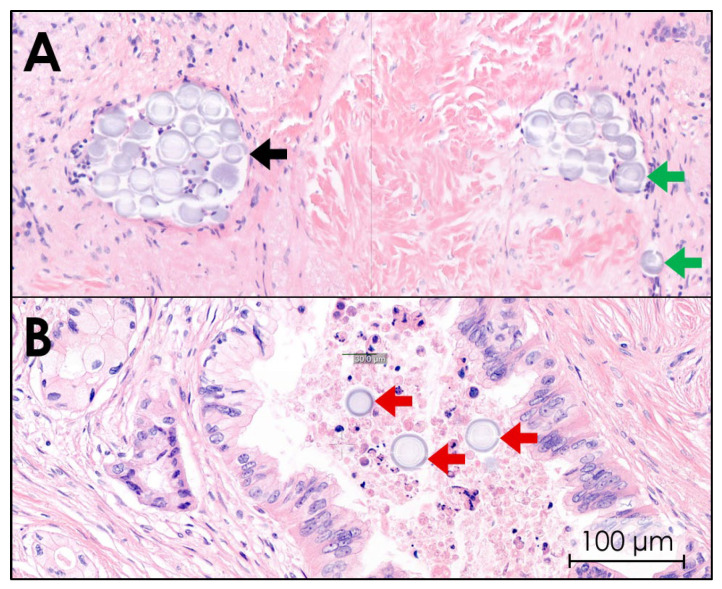
Hematoxylin–eosin staining of pancreatic cancer tissue after intratumoral holmium-165 microsphere (^165^HoMS) injection. Herein, purple-stained cell nuclei and gray–purple-stained ^165^HoMSs (±30 µm; circles) are visible. (**A**) Purple-stained tumor cell nuclei in a network of fibrosis (pink) with ^165^HoMS deposition in lymphatic lumen (black arrow) and interstitial lumen (green arrows). (**B**) ^165^HoMS deposition in a neoplastic duct (red arrows).

Histological images of all ten tissues confirmed that ^165^HoMSs lodged within the tumor. ^165^HoMSs were observed to have lodged within existing lumen, such as minor blood vessels and neoplastic ducts, and also in interstitial lumen newly formed by the injection pressure (see Figure 8). Large, empty, newly formed interstitial lumen were also observed and suggest that some of the ^165^HoMSs were washed out of the samples during the multiple tissue slicing, staining, and washing steps. Therefore, proper distribution patterns could not be established via this method.

## 4. Discussion

Minimally invasive therapies for downstaging or reducing the tumor burden of PDAC, especially in patients with LAPC, remain limited. Radioactive holmium-166 microspheres administered through intratumoral injection present a promising therapeutic option for this patient population. This study aimed to develop an intratumoral ^165^HoMS injection method in ex vivo human PDAC samples and to optimize injection parameters by using a novel injection system designed for ^165^HoMS suspension homogenization. These findings provide a foundation for future studies exploring intratumoral ^166^HoMS injection as a minimally invasive treatment approach for PDAC.

The injection system demonstrated promising results during both validation testing and intratumoral injection in ex vivo PDAC samples. For the ISPs, a ^165^HoMS syringe of 3.0 mL was optimal, balancing control over the injection volume with sufficient capacity. Pre-injection homogenization was achieved by rotating the syringe for at least 2 min, which was confirmed visually and through validation testing. Homogeneity was effectively maintained by rotating the syringe for at least 10 s between injections or after every 0.5 mL during consecutive injections. A rotation speed of 60 RPM minimized suspension idleness and could be comfortably sustained by hand for several minutes. An injection speed of 0.2 mL/s was identified as optimal for volume control while ensuring suspension homogeneity. To achieve a constant ^165^HoMS concentration, a minimum injection volume of 0.3 mL was required without an extension tube, and 0.5 mL was needed with an extension tube attached.

These parameters, along with the finalized injection system, resulted in a more constant injection of the ^165^HoMS concentration (R_HoMS−injection_) compared with previous injection techniques. In a study of head and neck squamous cell carcinoma, the injected ^166^HoMS concentrations ranged between 17.8 and 84.3%, whereas this study achieved a range of 69% to 93% [23]. In feline oral squamous cell carcinoma, the mean injected ^166^HoMS concentration reported was 59.8%, with a standard deviation (SD) of ±17.6%, whereas it was 81% with an SD of ±7.3% in this study [22]. Importantly, these comparisons involve both in vivo and laboratory settings (further discussed below). Nevertheless, the injection system and ISPs appear to enhance the ^165^HoMS injection efficiency while reducing the overall variation. The validated injection system and ISPs have been tested independently of PDAC, allowing their potential application in research involving intratumoral injections in animal models or other solid tumor types as a versatile platform technique [12].

A key consideration for the feasibility of intratumoral injection in PDAC was whether the exceptionally high interstitial pressure associated with this cancer type would permit fluid injections at all [25]. This elevated interstitial pressure is attributed to a dense extracellular matrix rich in activated fibroblasts and hyaluronan [25]. High interstitial pressure results in injection resistance, which can be sensed through haptic feedback from the syringe plunger. Accurately estimating interstitial pressures is crucial for ensuring safe intratumoral ^165^HoMS injection.

When considering the number of deposits injected per tumor, dividing the injection volume does not completely prevent leakage and introduces risks such as needle overshoot and increased tissue trauma. However, in tumors exceeding 10.0 mL, administering double or triple fractionated deposits may increase tumor dose coverage and minimize local saturation, thereby reducing the risk of unexpected fluid leakage. While not experimentally validated, it is recommended to limit the injection volume per deposit to a maximum of 1.0 mL to mitigate the risk of unpredictable distribution patterns. Experimental results indicate that deposit volumes of ≤1.0 mL result in localized deposition of ^165^HoMSs as presented in Figure 5, demonstrating a feasible degree of spatial control. Overall, overcoming interstitial pressure and effectively controlling intratumoral ^165^HoMS injections in ex vivo PDAC via the developed injection method, including the recommended ISPs and IIPs shown in Table 2 and Table 3, respectively, is feasible. Disregarding these parameters could result in the loss of ^165^HoMS concentration control or overall loss of injection feasibility.

Although multiple intratumoral injection therapies have been studied, detailed descriptions of injection parameters and techniques are scarce [11]. The in vivo intratumoral injection of ^32^P microparticles, which can be delivered either percutaneously [26,27,28] or via EUS [14,29], serves as the most comparable approach to the injection of ^165^HoMSs. The OncoPaC-1 trial, which employs intratumoral injection parameters similar to those established in this study, notably differs in the maximum injection volume (8% in OncoPaC-1 vs. 20% advised in this study) and only applies a single deposition per tumor [14]. A smaller injection volume might result in lower leakage risks and a lower tumor coverage; however, the results of the OncoPac-1 trial might also suggest that complete tumor coverage is unnecessary to establish a sufficient immunologic response and increase patient outcomes [14].

This study has several limitations regarding the generalizability of the results. The investigation involved a small sample size with significant variation in PDAC characteristics and with limited selection of patient demographics, including tumor morphology, location, intratumoral heterogeneity, vascularization, comorbidities, and prior anticancer treatment. Injection feasibility may vary among these factors, for example, due to changes in the tumor microenvironment following chemotherapy, which can differ between responders and nonresponders [30]. In addition to sample variation, the injection methodology and distribution evaluation also varied due to the exploratory and iterative design of this study, preventing further statistical analysis. Nuclear imaging with SPECT/CT is the standardized modality for observing ^166^HoMS distributions in humans [31] but is unavailable for nonradioactive ^165^HoMSs; thus, other methods (CT, MRI, US, pathology) were explored for further distribution assessment.

When IIPs are applied in vivo, additional factors such as vascularity, blood flow, and surrounding tissues may alter interstitial pressure, impacting feasibility. Thus, it should be recognized that the ex vivo results observed in this study may change when extrapolated to in vivo intratumoral injections. Further in vivo studies in human patients or animal models are necessary to validate these findings. Given the inherent variability of PDAC tumors within the patient population, specific injection limitations for guaranteed feasibility may not exist at all, emphasizing the need for a more patient-specific and image-guided approach. Thus, while the identified IIPs and cutoff values can be used as guidelines and may provide a foundation for future studies exploring intratumoral ^166^HoMS injections in PDAC, they may not be generalizable for all PDAC tumors, nor can they be directly extrapolated to an in vivo setting without further investigation.

In future investigations, several factors should be considered. High interstitial pressure plays a critical role in injection feasibility, necessitating assessment in an in vivo setting with a patient-specific methodology. The approach is also essential; options such as endoscopic ultrasound (EUS), open surgery, or percutaneous image-guided techniques are currently viable [11]. EUS guidance offers superior real-time visualization of the tumor and needle, although the visibility of the microspheres and the degrees of freedom for needle insertion are limited. While the ultrasound-guided approach utilized in this study could translate well to open surgical methods, surgery remains highly invasive, contradicting the goal of minimally invasive microbrachytherapy. Thus, a percutaneous, periprocedural image-guided approach might be the superior choice. Owing to the multimodality imaging characteristics of ^166^HoMSs, therapy guidance enables quantification of the ^166^HoMS distribution and further optimizes a patient-specific approach. Periprocedural imaging can be performed with MRI [18]; however, factors such as cost, acquisition speed, availability, standardized needle guidance, resolution, and magnetic restrictions favor CT guidance. Moreover, the clear visibility of the main ^165^HoMS deposits on CT in this study substantiate the use of CT guidance for both needle and therapy guidance. Still, post-injection validation by standardized SPECT/CT is necessary for confirmation of extratumoral and intratumoral distributions and for validation of novel dosimetry modalities. With respect to the target population, PDAC is known for its rapid systemic progression, meaning that a local therapy should always be combined with systemic anticancer treatment, such as conventional FOLFIRINOX chemotherapy [32]. Patients who may benefit most from enhanced local tumor control and potentially even from downstaging are the patients diagnosed with irresectable LAPC and who are receiving, or have just finished, systemic chemotherapy.

## 5. Conclusions

A feasible method was developed for intratumoral injection of holmium-165 microspheres in ex vivo pancreatic ductal adenocarcinoma. Adherence to the developed injection methods and guidelines is recommended for effective intratumoral deposition and minimal extratumoral leakage. The injection system and parameters developed here provide a foundation for future studies on holmium-166 microsphere injections in pancreatic cancer patients, with the aim to improve local tumor control and potentially broaden treatment options.

## Figures and Tables

**Table 1:** Microbrachytherapy agents studied for injection in pancreatic ductal adenocarcinoma.

Characteristic	Holmium-166 Microsphere	Phosphorus-32 Microparticle
Material	Poly L-lactic acid	Silicon
Diameter	30 µm (15–60 µm)	30 µm (15–50 µm)
Isotope	Holmium-166	Phosphorus-32
Half-life	26.8 h	14.27 days
Emission type (yield)	Beta (93.3%), gamma (6.7%)	Beta (100%)
Maximum beta energy	1.85 MeV	1.711 MeV
Mean β^−^ penetration	2.5 mm	2.76 mm
Maximum β^−^ penetration	8.7 mm	8.2 mm
Imaging modalities	SPECT, CT, MRI	Bremsstrahlung SPECT

**Table 2:** Established and laboratory-validated injection system parameters (ISPs). These parameters were used to establish a homogeneous and constant holmium-165 microsphere concentration during injection.

Parameter	Limit	Unit
Syringe volume	3.0	mL
Minimal rotation time	120	seconds
Intermittent rotation time ^1^	10	seconds
Rotation speed	60	RPM
Minimal flush volume	1.0	mL
Minimal injection volume without tube	0.3	mL
Minimal injection volume with tube	0.5	mL
Injection speed	0.2	mL/s

^1^ The minimal rotation time required between succeeding injections or after every 0.5 mL injected.

**Table 3:** Intratumoral injection parameters (IIPs) that were established after intratumoral holmium-165 microsphere injection in ex vivo pancreatic ductal adenocarcinoma samples.

Parameter	Limit	Unit
Tumor volume ^1^	2.5–15.6	mL
Maximum injection volume	20.0	% of tumor volume
Maximum syringe angle to horizontal	10.0	Degrees
Number of needle insertions	1–3	-
Number of deposits	1–3	-
Volume per deposit ^1^	0.3–1.0	mL
Holmium microsphere concentration in syringe ^1^	5.0–50.0	mg/mL
Needle diameter ^1^	18–21	Gauge
Needle length ^1^	50–150	mm

^1^ Parameters outside this range (one- or two-sided) were not applied in this study.

**Table 4:** Overview of tissue samples 1–10, intratumoral injection parameters, and observations.

Characteristics	Unit	Sample 1	Sample 2	Sample 3	Sample 4	Sample 5	Sample 6	Sample 7	Sample 8	Sample 9	Sample 10	
Tumor volume	mL	14.3	6.7	9.6	2.5	15.6	3.6	5.6	11.6	7.1	2.5	
Extratumoral leakage observed	Yes/no	yes	yes	no	no	no	yes	no	no	yes	yes	
Observation remark	-	Over fractionated deposits	Needle overshoot	-	HVC ^2^ instead of injection fluid	-	Intentionally increased injection volume	Needle blockage due to syringe angle	-	Leakage through pancreatic duct	Intentionally increased injection volume	
Image guidance	-	-	-	US	US	US	US + CT	US + CT	US + CT	US + CT	US + CT	
Injection volume (as % of tumor volume)	mL (%)	4.5 (31)	2.3 (35)	1.8 (19)	0.2 (8)	3.0 (19)	3.0 (83)	0.7 (13)	2.0 (17)	0.7 (10)	1.0 (40)	
Number of needle insertions	-	13	5	3	1	3	1	1	2	1	2	
Number of deposits × volume per deposit	mL	45 × 0.1	3 × 0.5 2 × 0.3	1 × 0.8 2 × 0.5	1 × 0.2	3 × 1.0	6 × 0.5	1 × 0.7	2 × 1.0	1 × 0.7	2 × 0.5	
Concentration of ^165^HoMSs in syringe	mg/mL	5.0	5.0	5.0	10.0	10.0	10.0	20.0	20.0	25.0	25.0	50.0
HoMSs injected ^1^	mg (mg/mL)	18.5 (4.1)	9.4 (4.1)	7.4 (4.1)	2.0 (10.0) ^2^	24.6 (8.2)	24.6 (8.2)	11.5 (16.4)	32.8 (16.4)	13.4 (20.5)	10.3 (20.5)	20.5 (41.0)

^1^ R_HoMS-injection_ was applied, which assumes 81% of the prepared ^165^HoMS concentration was injected, as determined during validation testing. ^2^ High-viscosity compound (HVC) was used instead of the injection fluid. For HVC, an R_HoMS_ of 100% was assumed since it does not suffer from accumulation within the injection system. HVC is currently not available in standard care.

## Data Availability

The data generated in this study can be made available upon reasonable request to the corresponding author.

## Supplementary Materials

The following supporting information can be downloaded at: https://www.mdpi.com/article/10.3390/cancers17061028/s1, Table S1: Imaging parameters.

## Appendix A. Extensive Injection System Development Results

An elaborated description of the established injection system parameters and findings during initial laboratory testing are described here.

1. Syringe volume: 3.0 mL (range: 1.0–10.0 mL)
  Syringe volumes of 1.0 mL resulted in less homogeneous suspensions and faster agglomeration, whereas syringes up to 10.0 mL resulted in excessive variation in the volume injected by manual injection due to the larger diameter.
2. Rotation time: 120 s (range: ~1–120 s)
  Visual homogenization of the ^165^HoMS suspension was achieved after 120 s in all the cases, even if the ^165^HoMSs accumulated to one side of the syringe before rotation. There were no clear differences in injection concentrations when rotating over 120 s.
3. Rotation speed: 60 RPM (range: 30–120 RPM)
  Since rotation of the syringe was performed manually, a rotation speed was evaluated which could be achieved for multiple minutes without exhausting the operator and did not cause visual agglomeration. Rotation speeds below 60 RPM caused moments of idleness, which resulted in agglomeration. When the suspension was injected, rotation of the syringe was not possible, and agglomeration of the suspension occurred within seconds. Therefore, between every separate injection, or every 0.5 mL of injection volume, the syringe was rotated again for at least 10 s.
4. Injection speed: 0.2 mL/s (range: 0.05–1.0 mL/s)
  Faster injection up to 1.0 mL/s could cause volume overshoots and thus increase the chance of leakage. Since the syringe could not be rotated during injection, lower injection speeds caused more ^165^HoMS agglomeration and more injection pauses to homogenize the suspension again.
5. Injection volumes: 0.3–1.0 mL (range: 0.1–1.0 mL)
  Because the injection system contains a dead volume, which is the lumen volume between the syringe exit and needle tip in which the ^165^HoMSs agglomerate, a minimal injection volume of 0.3 mL was used to ensure complete flow through the dead volume, and 0.5 mL when the plastic extension tube was attached.
6. System component diameters:
  System components with adjacent internal diameter changes, especially downstream narrowing, showed increased visual agglomeration of the ^165^HoMSs. Additionally, relatively large dead volumes, commonly found near Luer-lock connections, showed increased agglomeration when in a horizontal position. ^165^HoMS agglomeration within dead volumes caused a lower ^165^HoMS concentration; however, it could unexpectedly dislodge during injection and therefore suddenly increase the injected ^165^HoMS concentration.
