# Supplementary material for: Development of an Intratumoral Holmium Microsphere Injection Method in Ex Vivo Human Pancreatic Ductal Adenocarcinoma: A Preclinical Feasibility Study
Source: Cancers (Basel). 2025 Mar 19;17(6):1028. doi: 10.3390/cancers17061028 (PMC11941289; doi:10.3390/cancers17061028)
Supplement: Supplementary file 1 [file cancers-17-01028-s001.zip › cancers-3446790-supplementary.pdf]

Supplementary Materials: Development of an Intratumoral Holmium Microsphere Injection Method in *Ex Vivo* Human Pancreatic Ductal Adenocarcinoma: A Preclinical Feasibility Study

C. Ysbrand Willink, Sjoerd F.M. Jenniskens, Nienke J.M. Klaassen, Martijn W.J. Stommel, Cornelis J.H.M. van Laarhoven, Jurgen J. Fütterer and J. Frank W. Nijsen

Table S1. Imaging parameters.

**Brand**	**Siemens, Skyra**	
**Modality**	3 Tesla MRI	
**Sequence**	T1 vibe Dixon	T2 star flash (multi gradient echo)
**Coil**	Body coil	Body coil
**Resolution (mm)**	1.2 x 1.2	3.0 x 3.0<br>2.0 x 2.0
**Slice thickness (mm)**	3.0	3.5<br>4.0
**FoV (mm)**	308 x 380	384 x 384
**Repetition time (ms)**	4.0	188
**Number of echo's**	2	9
**Echo times (ms)**	1.3, 2.5	1.1 – 10.1
**Number of averages**	1	1
**Pixel bandwidth**	1040	1565
**Flip angle (degrees)**	9	37

**Brand**		**Canon, Aquilion**	
**Modality**	Clinical CT	**Filter material**	Aluminum/Copper
**Acquisition type**	Spiral	**Rescale Intercept**	0
**Spiral pitch factor**	0.813	**Rescale Slope**	1
**Samples per pixel**	1	**Kilo voltage peak (kVp)**	120
**Resolution (mm)**	0.5*0.5	**Exposure time (ms)**	600
**Slice thickness (mm)**	0.5	**Tube current (mA)**	300
**Reconstruction diameter (mm)**	320	**Exposure (mAs)**	180
**Reconstruction Algorithm**	Advanced intelligent Clear-IQ Engine (AiCE)	**Single collimation Width**	0.5
**Convolution Kernel**	BODY_SHARP	**Total collimation width**	40
**Image filter**	STD/BHC (standard dose/beam hardening correction)	**Pixel spacing**	0.5
**Filter type**	MEDIUM		
**Focal Spot(s)**	0.9/0.8		

MRI and CT imaging parameters utilized during this study.
